# #KneeOsteotomy: Evaluation of the Quality and Educational Value of TikTok Videos

**DOI:** 10.1177/23259671261428460

**Published:** 2026-05-04

**Authors:** Moses K.D. El Kayali, Luis V. Bürck, Stephen Fahy, Alan Getgood, Benjamin Bartek, Tobias Jung, Stephan Oehme

**Affiliations:** † Charité–Universitätsmedizin Berlin, Center for Musculoskeletal Surgery, Berlin, Germany; ‡ Sporthopaedicum Berlin, Berlin, Germany; § ASPETAR, Orthopaedic and Sports Medicine Hospital, Doha, Qatar; ‖ Berlin Institute of Health at Charité–Universitätsmedizin Berlin, Julius Wolff Institute, Berlin, Germany; Investigation performed at Charité–Universita « tsmedizin Berlin, Center for Musculoskeletal Surgery, Berlin, Germany

**Keywords:** knee osteotomy, TikTok, social media, high tibial osteotomy, distal femoral osteotomy, patient education

## Abstract

**Background:**

TikTok videos on orthopaedic topics receive high engagement, but the quality of content on knee osteotomies is unclear.

**Purpose:**

To assess the quality, reliability, and educational value of TikTok videos on knee osteotomy.

**Study Design:**

Cross-sectional study.

**Methods:**

TikTok was searched for “knee osteotomy,”“high tibial osteotomy,” and “distal femoral osteotomy,” yielding 789 videos. A total of 191 met inclusion criteria. Video metrics (duration, views, likes, shares), uploader type (private user, physical therapist, physician, researcher), and content type (patient experiences, physical therapy and rehabilitation, anatomy, surgical technique) were recorded. Quality was assessed using the DISCERN instrument, *Journal of the American Medical Association* (*JAMA*) benchmark criteria, and Global Quality Score (GQS). Associations between video metrics and quality scores were analyzed using Spearman rank correlation, and Mann-Whitney *U* tests evaluated differences in scores by uploader type and content type.

**Results:**

Most videos were posted by private users (145; 75.9%) and focused on patient experiences (128; 67.0%). Mean duration was 34.4 ± 40.8 seconds (range, 4-317 seconds). Videos received a mean of 3122.3 ± 16,845.1 likes (range, 0-197,400 likes), 223.9 ± 1891.3 shares (range, 0-25,900 shares), and 166,863.0 ± 1,002,969.9 views (range, 70-12,700,000 views). Mean DISCERN, *JAMA*, and GQS scores were 32.1 ± 18.4, 1.7 ± 1.1, and 2.6 ± 0.9. Video duration, shares, and views correlated with all quality scores (*P* < .05), while likes correlated weakly with DISCERN only (*P* < .05). Videos from health care professionals (physicians, physical therapists, researchers) achieved significantly higher quality scores than private users (DISCERN 56.0 ± 14.2 vs. 24.5 ± 11.9; GQS 3.6 ± 0.8 vs. 2.2 ± 0.5; *JAMA* 3.2 ± 1.0 vs. 1.3 ± 0.7; all *p* < 0.001). Educational videos (anatomy, physical therapy/rehabilitation, surgical technique) scored significantly higher quality scores than patient experience videos (DISCERN, 52.8 ± 16.8 vs 21.9 ± 6.9; GQS, 3.6 ± 1.0 vs 2.1 ± 0.3; *JAMA*, 2.9 ± 1.2 vs 1.1 ± 0.3; all *P* < .001).

**Conclusion:**

TikTok videos related to knee osteotomy demonstrated overall low quality. Although videos produced by health care professionals achieved higher quality scores, overall content quality remained limited.

Knee osteotomy comprises a group of surgical procedures designed to redistribute load-bearing forces within the knee joint.^24,31^ The most commonly performed techniques include high tibial osteotomy, distal femoral osteotomy, and slope-correcting osteotomies.^7,16,18,34,43^ These procedures are primarily indicated for the treatment of unicompartmental osteoarthritis associated with malalignment, for the management of failed knee ligament reconstructions, persistent instability, or protecting cartilage-related procedures.^1,15,24,26,27,37^ When appropriately indicated and executed, knee osteotomies have demonstrated favorable outcomes, with reliable improvements in pain, function, and joint longevity.^3,17,20,22^

Over the past decade, social media (SM) has emerged as a dominant medium for information dissemination, including health-related content. More than 90% of American teenagers actively use SM, and approximately 42% of Americans report turning to SM platforms for health information.^41,54^ These platforms allow individuals, particularly those with limited access to health care, to seek guidance without direct interaction with a medical professional.^50^

Despite this potential benefit, studies indicate that only a small fraction of medically oriented SM content is produced by board-certified professionals.^6,38,51,60^ Consequently, the vast majority of health information shared online bypasses traditional peer-review mechanisms, raising concerns regarding its accuracy, quality, and reliability.^2,4,6,12,13,33,44^ This concern is particularly relevant for rapidly growing platforms such as TikTok (ByteDance Ltd), where medical information is widely disseminated yet largely unregulated. While the quality of orthopaedic content on platforms such as YouTube (Google LLC) has been investigated, TikTok, despite its meteoric rise, remains underexplored in this context.^11,14,30,35,45^ TikTok, launched in 2016, has become one of the fastest-growing SM applications worldwide with >1.6 billion active users and >5 billion downloads in 2023, largely driven by its short form video format and algorithm-based content delivery that promotes rapid dissemination and high user engagement.^55^ Given TikTok's popularity and influence, understanding the quality of medical content on this platform is critical, particularly for specialized procedures such as knee osteotomy, which is increasingly and successfully performed in young, active patients and requires careful patient education and informed decision-making.^19,27,58,59^

The purpose of this study was to evaluate the quality, reliability, and educational value of TikTok videos related to knee osteotomy. We hypothesized that TikTok content on this topic would demonstrate low quality, limited reliability, and minimal educational value and that the proportion of videos created by health care professionals would be low.

## Methods

### Search Strategy and Video Selection

As this study analyzed publicly available SM content without involving human participants, institutional review board approval was not required.

The SM platform TikTok (https://www.tiktok.com) was searched on July 25, 2025, to identify videos related to knee osteotomy procedures. The keywords used were “knee osteotomy,”“high tibial osteotomy,” and “distal femoral osteotomy.” The search aimed to replicate the typical experience of a layperson seeking knee osteotomy information.

**Figure 1.**
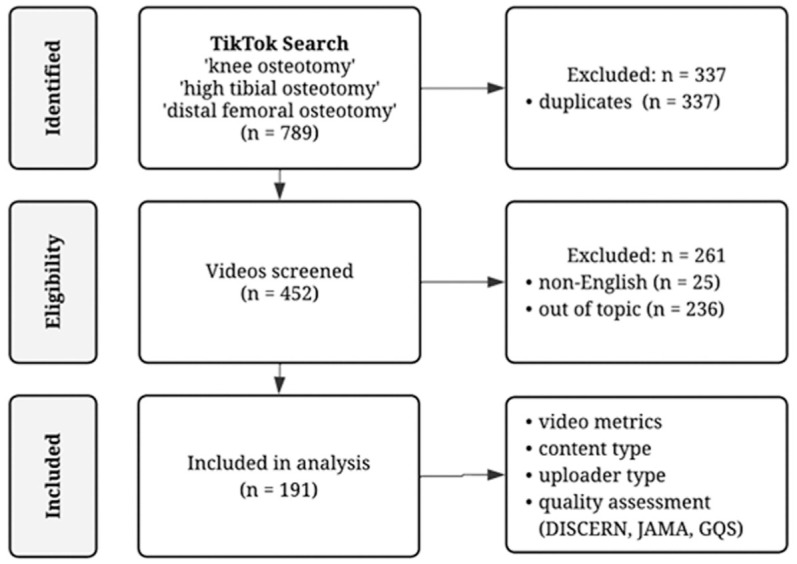
Flowchart of video selection process performed in this study. DISCERN, DISCERN questionnaire; GQS, Global Quality Score; *JAMA*, *Journal of the American Medical Association*.

A total of 789 videos were initially retrieved and screened for eligibility. Videos were excluded if they were duplicates, non-English, or unrelated to knee osteotomy procedures. The inclusion process is summarized in Figure 1.

### Data Collection and Video Classification

For each included video, the following information was recorded: video metrics (duration [seconds], number of views, likes, and shares); uploader type (private user, physical therapist, physician, or researcher), content type (patient experiences, physical therapy and rehabilitation, anatomy, or surgical technique). Uploader type was determined from the TikTok account profile and associated content. Private users were defined as accounts without professional credentials in the username, bio, or videos, and without affiliation to a medical or academic institution. Physical therapists were identified by credentials (eg, PT, Physiotherapist) or clear association with physical therapy services. Physicians were identified by medical degrees (eg, MD, DO), stated specialty, or affiliation with a health care institution. Researchers were identified by affiliation with a university, research group, or laboratory or by research-focused content without clinical practice credentials.

### Assessment of Video Reliability and Quality

All videos were independently assessed by 2 board-certified orthopaedic surgeons (S.F. and S.O.), both with clinical and academic experience in knee surgery. Prior to formal evaluation, both reviewers reviewed the original descriptions and scoring instructions for the assessment tools to ensure consistent application. Videos were evaluated independently and in random order. Reviewers were blinded to uploader identity, account type, video description, and profile information, and assessments were based solely on video content. Interrater reliability was assessed by calculating the intraclass correlation coefficient (ICC).^48^ In cases of discrepant ratings, a consensus score was determined through joint re-review and discussion.

Three validated assessment tools were applied: the DISCERN questionnaire, the *Journal of the American Medical Association* (*JAMA*) benchmark criteria, and the Global Quality Score (GQS), which have been widely used in prior analyses of online health content.^5,6,9,13,39,44,49,56^

### DISCERN Questionnaire

The DISCERN instrument (https://www.discern.org.uk) is a 16-item tool to assess reliability and quality of health-related information.^9^ It is divided into 3 sections: items 1 to 8 assess reliability, items 9 to 15 focus on the quality of treatment information, and item 16 provides an overall quality rating. Items 1 to 15 are scored on a 5-point scale (1 = inadequate; 5 = fully adequate), resulting in a total score between 15 and 80. Item 16 serves as a global rating, where 1 indicates very poor quality with major deficiencies and 5 indicates excellent quality with minimal deficiencies. The total DISCERN score is calculated as the sum of the first 15 items, ranging from 15 to 75. Higher scores indicate greater reliability and quality of information. Scores are interpreted as follows: 15 to 27, very poor; 28 to 38, poor; 39 to 50, medium; 51 to 62, good; and 63 to 75, excellent.^6,13^

### *JAMA* Benchmark Criteria

The *JAMA* benchmark criteria evaluate 4 domains of online medical content, authorship, attribution, disclosure, and currency, scoring 0 to 4.^49^ Each fulfilled criterion is awarded 1 point, resulting in a total score from 0 to 4. Scores of 0 to 1 indicate insufficient information, 2 to 3 reflect partially sufficient information, and 4 represents fully sufficient information. Higher scores reflect greater transparency and accountability of the information.

### Global Quality Score

The GQS rates the overall quality and usefulness of online videos on a 5-point scale, where 1 indicates poor and 5 indicates excellent quality.^5^ Unlike DISCERN and *JAMA*, which focus on reliability, transparency, and treatment-specific information, the GQS reflects the reviewer's global impression of content quality from a patient education perspective.

### Statistical Analysis

All extracted data were organized in Microsoft Excel (Version 16.78; Microsoft Corp) and analyzed using IBM SPSS Statistics (Version 28.0; IBM Corp). Descriptive statistics were reported as mean ± SD and range. For nonparametric comparisons, medians and interquartile ranges were additionally reported. The Shapiro-Wilk test assessed normality, and Levene test evaluated homogeneity of variances.

Videos were additionally categorized, with DISCERN scores ≥51 considered good or excellent quality, *JAMA* scores ≥2 indicating at least partially sufficient transparency, and GQS scores ≥4 reflecting high quality. Spearman rank correlation was applied to examine associations between video metrics and the DISCERN, *JAMA*, and GQS scores, with correlation coefficients (*r*) and *P* values reported. A correlation coefficient of 0.00 to 0.19 was considered very weak, 0.20 to 0.39 weak, 0.40 to 0.59 moderate, 0.60 to 0.79 strong, and 0.80 to 1.0 very strong.^46^ Differences in score by uploader type and content type were assessed with the Mann-Whitney *U* test. Because of small subgroup sizes, physical therapists, physicians, and researchers were grouped as “health care professionals,” and anatomy, physical therapy, and surgical technique videos were grouped as “educational content.” All tests were 2-tailed, and *P* < .05 was considered statistically significant. Where applicable, Bonferroni correction was applied to adjust for multiple comparisons. ICCs were interpreted as follows: slight (0.00-0.20), fair (0.21-0.40), moderate (0.41-0.60), good (0.61-0.80), or excellent (>0.8).^25^

## Results

A total of 191 TikTok videos related to knee osteotomy were included in the analysis. Of these, 145 (75.9%) were posted by private users, 31 (16.2%) by physicians, 9 (4.7%) by physical therapists, and 6 (3.1%) by researchers. In terms of content, 128 videos (67.0%) presented patient experiences, 32 (16.8%) demonstrated surgical techniques, 17 (8.9%) focused on physical therapy, and 14 (7.3%) addressed anatomy.

The mean video duration was 34.4 ± 40.8 seconds (range, 4-317 seconds). Videos received a mean of 3122.3 ± 16,845.1 likes (range, 0-197,400 likes), 223.9 ± 1891.3 shares (range, 0-25,900 shares), and 166,863.0 ± 1,002,969.9 views (range, 70-12,700,000 views). When stratified by uploader type, videos posted by health care professionals had a mean duration of 40.8 ± 41.4 seconds (range, 7-245 seconds) and received a mean of 5515.7 ± 29,068.7 likes (range, 1-197,400 likes), 664.4 ± 3807.6 shares (range, 0-25,900 shares), and 80,794.5 ± 186,697.8 views (range, 434-860,417 views). Videos uploaded by private users had a mean duration of 32.4 ± 40.5 seconds (range, 4-317 seconds), with a mean of 2363.0 ± 10,389.0 likes (range, 0-68,438 likes), 84.2 ± 327.5 shares (range, 0-2490 shares), and 62,218.9 ± 321,909.0 views (range, 70-3,600,000 views).

Interrater reliability was excellent for all scores (ICC > 0.8). The overall mean DISCERN score was 32.1 ± 18.4 (range, 16-76). The *JAMA* score was 1.7 ± 1.1 (range, 1-4), and the mean GQS was 2.6 ± 0.9 (range, 2-5). Overall, 44 videos (23.0%) achieved good or excellent quality based on DISCERN scores, 68 videos (35.6%) demonstrated at least partially sufficient transparency according to *JAMA* criteria, and 41 videos (21.5%) were rated as having high quality based on GQS scores.

Spearman correlation analysis revealed that video duration was positively correlated with all 3 quality scores (DISCERN: *r* = 0.274, *P* < .001; GQS: *r* = 0.316, *P* < .001; *JAMA*: *r* = 0.288, *P* < .001). Number of likes correlated weakly with DISCERN only (*r* = 0.152; *P* = .04). Number of shares correlated positively with all 3 scores (DISCERN: *r* = 0.259, *P* < .001; GQS: *r* = 0.246, *P* < .001; *JAMA*: *r* = 0.223, *P* = .002), and number of views showed weak but significant correlation with all scores (DISCERN: *r* = 0.184, *P* = .01; GQS: *r* = 0.159, *P* = .03; *JAMA*: *r* = 0.153, *P* = .03) (Table 1).

**Table 1:** Correlation Analysis Between Video Characteristics and Quality Scores*^*a*^*

Variables (based on total number)	DISCERN		*JAMA*		GQS	
	*r* *^*b*^*	*P*	*r* *^*b*^*	*P*	*r* *^*b*^*	*P*
Duration	0.274	**<.001**	0.316	**<.001**	0.288	**<.001**
Likes	0.152	**.04**	0.121	.10	0.115	.11
Shares	0.259	**<.001**	0.246	**<.001**	0.233	**.002**
Views	0.184	**.01**	0.159	**.03**	0.153	**.03**

a Bold values indicate statistical significance at *P* < .05. DISCERN, DISCERN questionnaire; *JAMA*, *Journal of the American Medical Association*; GQS, Global Quality Score.

b Spearman rank correlation.

Videos by health care professionals achieved significantly higher DISCERN (*P* < .001), GQS (*P* < .001), and *JAMA* (*P* < .001) scores than those posted by private users (Table 2).

**Table 2:** Differences in Video Quality Scores by Uploader Type*^*a*^*

Score	Private Users (n = 145)	Health Care Professionals (n = 46)	*P*
DISCERN	24.5 ± 11.9; 21.0 (17.5-25.0)	56.0 ± 14.2; 65.0 (46.0-65.0)	**<.001**
*JAMA*	1.3 ± 0.7; 1.0 (1.0-1.0)	3.15 ± 1.0; 4.0 (2.0-4.0)	**<.001**
GQS	2.2 ± 0.5; 2.0 (2.0-2.0)	3.65 ± 0.8; 4.0 (3.0-4.0)	**<.001**

a Data are presented as mean ± SD; median (IQR). Bold *P* values indicate statistical significance at *P* < .05. Bonferroni correction applied. DISCERN, DISCERN questionnaire; GQS, Global Quality Score; *JAMA*, *Journal of the American Medical Association*.

Patient experience videos scored significantly lower than educational content for DISCERN (*P* < .001), GQS (*P* < .001), and *JAMA* (*P* < .001) (Table 3).

**Table 3:** Differences in Video Quality Scores by Content Type*^*a*^*

Score	Patient Experiences (n = 128)	Health Care Professionals (n = 63)	*P*
DISCERN	21.9 ± 6.9; 19.0 (17.0-25.0)	52.8 ± 16.8; 61.0 (38.0-65.0)	**<.001**
*JAMA*	1.1 ± 0.3; 1.0 (1.0-1.0)	2.9 ± 1.2; 3.0 (2.0-4.0)	**<.001**
GQS	2.1 ± 0.3; 2.0 (2.0-2.0)	3.6 ± 1.0; 4.0 (3.0-4.0)	**<.001**

a Data are presented as mean ± SD; median (IQR). Bold *P* values indicate statistical significance at *P* < .05. Bonferroni correction applied. DISCERN, DISCERN questionnaire; GQS, Global Quality Score; *JAMA*, *Journal of the American Medical Association*.

## Discussion

The key finding of this study was that TikTok content related to knee osteotomy demonstrated overall low quality and limited reliability, confirming our hypothesis. The mean DISCERN score was 32.1 ± 18.4 (poor), the mean *JAMA* score was 1.7 ± 1.1 (insufficient), and the mean GQS was 2.6 ± 0.9 (low educational value). Most videos (75.9%) were posted by private users, with only 24.1% produced by health care professionals. Videos produced by health care professionals achieved significantly higher DISCERN, GQS, and *JAMA* scores than those from private users (all *P* < .001). Similarly, educational content scored significantly higher than patient experience videos across all 3 scoring systems (all *P* < .001). This finding is partly explained by the predominance of private user videos centered on patient experiences, which are not designed to provide comprehensive educational information and therefore inherently score lower on the assessment tools. To our knowledge, this is the first study to evaluate TikTok content related to knee osteotomies.

Our findings are consistent with prior research evaluating orthopaedic content on TikTok. One study analyzed 111 TikTok videos related to anterior cruciate ligament (ACL) rehabilitation exercises using the hashtags “#ACLrehab” and “#ACLexercises.”^6^ Despite accumulating >5.5 million views, the videos, mostly created by private users, showed very poor educational value. Another study of 100 TikTok videos about the ACL similarly found that the overall quality was extremely low, with mean DISCERN, *JAMA*, and GQS all reflecting poor reliability.^13^ Interestingly, while prior studies have reported only modest differences between surgeon-produced and private user videos, health care professional videos in our study demonstrated clearly higher scores; however, overall quality remained limited. In line with these findings, another study examining 64 TikTok videos on total knee arthroplasty rehabilitation reported that no videos achieved an “excellent” rating, with most classified as “poor” even when created by health care professionals.^56^ Similarly, an assessment of the 100 most popular TikTok videos about knee osteoarthritis revealed that although 67.7% were uploaded by medical professionals, the strength of evidence supporting the recommendations was low, and no significant correlation existed between video engagement and evidence quality.^21^ In contrast to that, and supported by the findings of D’Ambrosi and Hewett^13^ video metrics such as number of views, likes, shares, and video length demonstrated statistically significant but predominantly weak correlations with ≥1 score in our study. The relatively low scores of health care professional videos likely reflect the short form, engagement-driven nature of TikTok, which limits comprehensive discussion of indications, risk, and references, as well as the mismatch between brief video formats and assessment tools designed for more detailed educational content.

Similarly, TikTok content on other musculoskeletal topics, including scoliosis, Achilles tendinopathy, hip fractures, frozen shoulder, and osteoporosis demonstrates consistently low reliability, quality, and educational value.^8,10,28,29,53^ Collectively, these studies demonstrate that while TikTok garners substantial patient engagement on orthopaedic topics, the educational quality and adherence to evidence-based guidelines remain poor, highlighting the platform's risk for disseminating medical misinformation and an opportunity for orthopaedic specialists to provide higher-quality digital education.

A recent cross-platform analysis of 130 posts on knee osteoarthritis across TikTok, Instagram (Meta Platforms Inc), and YouTube confirmed that low content quality is not unique to TikTok, though TikTok performed the worst despite achieving the highest user engagement.^21^ The inherently short form, engagement-driven format of TikTok, and its replication on other platforms through features such as Instagram Reels, Facebook Reels (Meta Platforms Inc) and YouTube Shorts, makes it difficult to provide comprehensive, evidence-based medical information. The focus on rapid viewer retention and algorithmic promotion of highly engaging content often prevents thorough discussion of indications, risks, benefits, and safety precautions and may contribute to echo chambers that amplify sensational or misleading health content.^52^ Moreover, like other SM platforms, TikTok lacks the scientific oversight and peer-reviewed mechanisms necessary to ensure the quality and reliability of medical content, and the implementation of a formal peer-reviewed system for health care–related TikTok videos is unlikely, given the platform's structure.^42^

Improving the accuracy of orthopaedic information on TikTok requires active engagement from health care professionals, who should be encouraged to contribute high-quality, evidence-based content to actively improve the quality and quantity of information available. Correcting misinformation can begin through commenting on existing videos with evidence-based clarifications and linking to peer-reviewed sources.^36^ Creating original, high-quality short form videos can also be an effective strategy, as demonstrated by the potential reach of orthopaedic content, with our study identifying a video with 12.7 million views on knee osteotomy.

Podcasts in orthopaedic surgery have already emerged as a successful educational tool for health care providers and trainees, serving as a complementary medium to traditional publications by quickly disseminating main findings and raising awareness of emerging research.^23,40,47^ Similarly, short form video content could replicate this success on platforms like TikTok by reaching patients and the general public with concise, evidence-based messages. These educational clips could answer common patient questions^6^ such as, “When can I return to sports after a knee osteotomy” or “When should I worry about my wound after surgery?,” as well as provide visual explanations of the surgical procedure, rehabilitation milestones, myth-busting content, lifestyle advice during recovery, and simplified summaries of recent research or evidence-based recommendations. Additionally, expert video assessments could be incorporated into platform algorithms to promote reliable content, which would further incentivize the production of peer-reviewed, evidence-based posts.^32^

As 57% of orthopaedic patients report using SM for health care information,^57^ it is important to encourage critical appraisal of online health information and to guide patients toward reliable sources, such as professional society resources and institutional websites. Rather than attempting to monitor or correct medical content across all SM platforms, a more sustainable approach may be to ensure that accurate, evidence-based orthopaedic information is accessible through trusted channels and to support patient education on how to evaluate online content. This perspective is further supported by evidence, including our findings, indicating that SM-based education often remains of limited quality even when produced by health care professionals.

### Limitations

This study has several limitations. First, it evaluated TikTok exclusively, whereas other SM platforms have different video formats, audiences, and content moderation policies, limiting the generalizability of our findings. We chose TikTok because it is the fastest-growing platform and represents the current trend toward short, fast-paced, and engagement-driven video content, which is increasingly shared across other platforms. Second, search and selection bias may have influenced video inclusion, as TikTok's algorithm is affected by location, personalization, and trending content. Moreover, the dynamic nature of SM means that our results represent only a snapshot in time, as video availability and trends change rapidly. Additionally, the time elapsed since video publication was not controlled for and may have influenced engagement metrics and visibility, which could affect correlations between video popularity and quality scores. Furthermore, this study did not include a qualitative thematic analysis, which may have provided additional insight into content characteristics driving user engagement. Third, only English-language videos were analyzed, which may not reflect the quality of content available in other languages. Fourth, we focused on a narrow and highly specific topic, aiming to simulate the search behavior of a typical user seeking information on knee osteotomies. While this limits the breadth of our analysis, our findings are consistent with prior orthopaedic and nonorthopaedic SM research demonstrating overall low content quality. Additionally, because many private user videos consisted of patient experience narratives rather than educational content, applying scoring tools designed to assess reliability and educational quality may inherently result in lower ratings, representing a potential source of selection bias. Finally, although we used standardized and widely accepted scoring tools for SM content evaluation, video assessment inherently involves a degree of subjectivity.

## Conclusion

TikTok videos related to knee osteotomy demonstrated low quality and limited reliability, with the majority produced by private users. Although videos created by health care professionals achieved significantly higher quality scores, overall content quality remained limited. Given the growing influence of SM on patient knowledge and decision-making, ensuring access to accurate, evidence-based orthopaedic information through trusted sources remains important.
